# An investigation into the interaction between water deficit and injury of sugarcane borer (Lepidoptera: Crambidae) in the gas exchange parameters and spectral reflectance of sugarcane

**DOI:** 10.1002/jsfa.70093

**Published:** 2025-07-31

**Authors:** João Rafael Silva Soares, Vinicius Cesarin, Claudiane Martins da Rocha, Júlia Karoline Mercês, Fernanda Sayuri Yoshino Watanabe, Nilton Nobuhiro Imai, Antonio Maria Garcia Tommaselli, Priscila Lupino Gratão, Odair Aparecido Fernandes

**Affiliations:** ^1^ São Paulo State University (Unesp), School of Agricultural and Veterinary Sciences Jaboticabal Brazil; ^2^ São Paulo State University (Unesp), School of Technology and Sciences Presidente Prudente Brazil

**Keywords:** stress combination, insect–plant interaction, decision making, gas exchange

## Abstract

**BACKGROUND:**

Plants are subject to a variety of stressors that will eventually limit their development and yield. However, analysis of the combination of abiotic and biotic stresses is usually disregarded. The combination of stresses can have synergistic or antagonistic effects and, therefore, they require greater clarification for assertive decision‐making. In this study, we examined how the interaction between water stress and sugarcane borer injury alters the physiological aspects of sugarcane through gas exchange parameters and foliar reflectance.

**RESULTS:**

Our results show that both stressors alter sugarcane physiology, but it is not possible to distinguish between the effects of sugarcane borer injury and those caused by water deficit based on the methodology adopted. Stresses, whether isolated or combined, disrupt gas exchange parameters, regardless of the post‐infestation period and sugarcane variety. Among the differences that affect the combination of stressors are transpiration, stomatal conductance, and CO_2_ assimilation. The intercellular CO_2_ concentration and water use efficiency presented a positive and negative relationship, respectively, with lesion size (gallery length). The spectral response of sugarcane plants was similar regardless of treatments, with low classification metrics from machine learning models.

**CONCLUSION:**

The interaction between water stress and sugarcane borer injury alters key physiological traits in sugarcane, although their individual effects could not be fully distinguished. The knowledge generated from this study supports the possibility of refining the economic thresholds for the sugarcane borer in sugarcane since changes in sugarcane tolerance imposed by water restrictions can alter the current threshold applied. © 2025 The Author(s). *Journal of the Science of Food and Agriculture* published by John Wiley & Sons Ltd on behalf of Society of Chemical Industry.

## INTRODUCTION

Abiotic and biotic stresses are the main limiting factors in plant growth and development. The impact of stressors on plants can vary depending on the severity and duration of stress, development stage, tissue type and interactions among multiple stresses.^1^, ^2^ For instance, the combination of abiotic and biotic stresses can change the signaling pathways of plant defense, either activating or suppressing defensive responses.^3^, ^4^, ^5^

The assessment of the impacts of abiotic and biotic stresses on plants has usually been carried out individually, ignoring the effects of their interactions.^6^, ^7^ This happens because, in general, dividing and quantifying the effects of multiple stressor interactions in plants are not easy tasks.^8^, ^9^, ^10^ However, plants in agroecosystems or in natural systems are often exposed to simultaneous abiotic and biotic stresses, which can significantly impact their development and yield. Additionally, the genetic differences between genotypes, the effects of abiotic and biotic stressors on plants and their interaction with the environment should all be considered when evaluating the quantitative and qualitative responses of plants to these types of stressors.^10^, ^11^, ^12^

Despite its rusticity among cultivated plants, sugarcane (*Saccharum* spp.) is also subject to several stressors that constrain its potential yield. Water restriction, for example, is considered the most limiting abiotic factor stress for sugarcane production.^13^ Under water restriction, plants conserve water to balance their use and consequently reduce CO_2_ capture, carbon fixing, and metabolic processes by closing the stomata.^1^, ^2^, ^13^ Stress also changes physiological signaling molecules like trehalose, hydrogen peroxide (H_2_O_2_), and malondialdehyde, and the activity of enzymes that detoxify free radicals, including superoxide dismutase (EC 1.15.1.1), catalase (EC 1.11.1.6), and ascorbate peroxidase (EC 1.11.1.11).^3^, ^4^, ^14^ These byproducts trigger response cascades, modifying metabolic and molecular pathways to defend the plant.^5^, ^15^

Many of the physiological and chemical properties of plants influence how their tissues reflect and absorb light.^16^ These properties can change under stress and alter the reflectance spectrum of the leaves.^17^ Advances in optical devices, such as hyperspectral sensors, offer greater potential for rapid stress detection by capturing hundreds of individual wavelengths or narrow wavebands of reflected electromagnetic energy from targets.^18^ Combined with machine learning tools, this information has accurately classified many instances of both abiotic and biotic stressors in plants.^17^, ^19^, ^20^

In recent decades, several studies have investigated the impact of water deficits on sugarcane genotypes and have provided important insights into the physiological responses of plants when facing this stress.^6^, ^13^ Understanding the mechanisms by which insect‐induced herbivory, in combination with other stressors, alters plant physiology is essential for improving pest monitoring programs, developing prediction models, making informed decisions to mitigate potential losses and even contributing to genetic breeding programs.^21^

The sugarcane borer (SB) *Diatraea saccharalis* (Fabricius, 1794) (Lepidoptera: Crambidae) is one of the main pests affecting sugarcane.^22^ The larvae damage sugarcane by boring holes and galleries in the culms, leading to yield loss and increasing plant susceptibility to fungal attack, which cause the red rot disease complex (*Colletotrichum* spp. and *Fusarium* spp.), as well as the death of the buds and physiological disorders in the lateral culm buds.^22^, ^23^ Occasionally, SB larvae bore circular galleries, weakening the culm and potentially causing plants to fall due to wind action. A SB attack during initial growth when plants do not have internodes developed causes central leaf yellowing, a symptom known as a ‘dead heart’. The SB associated with phytopathogenic fungi is responsible for average sugar yield losses ranging from 0.52% and 1.10% for every 1% of bored internodes.^23^

In the work reported here, we investigated how water stress and SB injury alter the physiological and spectral responses of sugarcane. It is hypothesized that injury caused by SB larvae triggers a series of morphophysiological responses in sugarcane plants. Due to the characteristic injury caused by the pest, it is assumed that SB injury blocks the passage of water and essential elements to the aerial parts of the plant and generates symptoms like those of a water deficit. However, we hypothesize that these stresses can be distinguished from each other.

## MATERIALS AND METHODS

### Plants

Pre‐sprouted sugarcane settlings of the varieties CT 022994 and CTC 4 were multiplied according to Landell *et al*.^24^ and transferred to plastic pots (22 L capacity) containing 23 kg of agricultural soil from the 0–30 cm profile to maintain the porous space and water retention capacity. The plants were kept in a greenhouse at 27 ± 3 °C, RH 70 ± 10% and natural light. An automatic irrigation system supplied daily water to the plants until the start of the experiment, 120 days after transplantation (DAT) of seedlings. To standardize the number of culms per pot, we removed less developed culms during the tillering phase (approximately at 50 DAT), ensuring that each pot had only one culm remaining.

### Experimental design

The experiment followed a randomized block design in the factorial scheme 2 × 2 × 2 (Table 1), i.e. two water regimes (NS = no water deficit; WS = water deficit), two infestation levels (C = control – no infestation; DS = SB infested) and two sugarcane varieties (CTC 4 and CT 022994). The selected varieties are widely cultivated in São Paulo State, Brazil, and were chosen based on their contrasting responses to water availability: CT 022994 is recognized as drought‐tolerant, while CTC 4 is considered drought‐susceptible. Importantly, both cultivars are known to be susceptible to SB attack.^25^

**Table 1:** Factorial experiment setting, where two sugarcane varieties were subjected to two irrigation regimes (no water stress (NS) *versus* water restriction (WS)) and the attack by SB (no SB infestation (C) *versus* SB infested)

Treatment	Variety	Water regime	Infestation
NSC	CT 022994	100% WRC	Control
NSDS	CT 022994	100% WRC	Infested
NSC	CTC 4	100% WRC	Control
NSDS	CTC 4	100% WRC	Infested
WSC	CT 022994	60% WRC	Control
WSDS	CT 022994	60% WRC	Infested
WSC	CTC 4	60% WRC	Control
WSDS	CTC 4	60% WRC	Infested

Each treatment had 20 pots, each of which was considered a replication and contained a single sugarcane plant, totaling 160 pots. To simulate water deficit, plants were subjected to a water regime of either 60% or 100% of soil water retention capacity (WRC), representing deficit and non‐deficit conditions, respectively. The water deficit was controlled by daily weighing of the pots, and water was replenished based on the average weight loss of 12 pots per treatment regardless of the variety.

**Figure 1:**
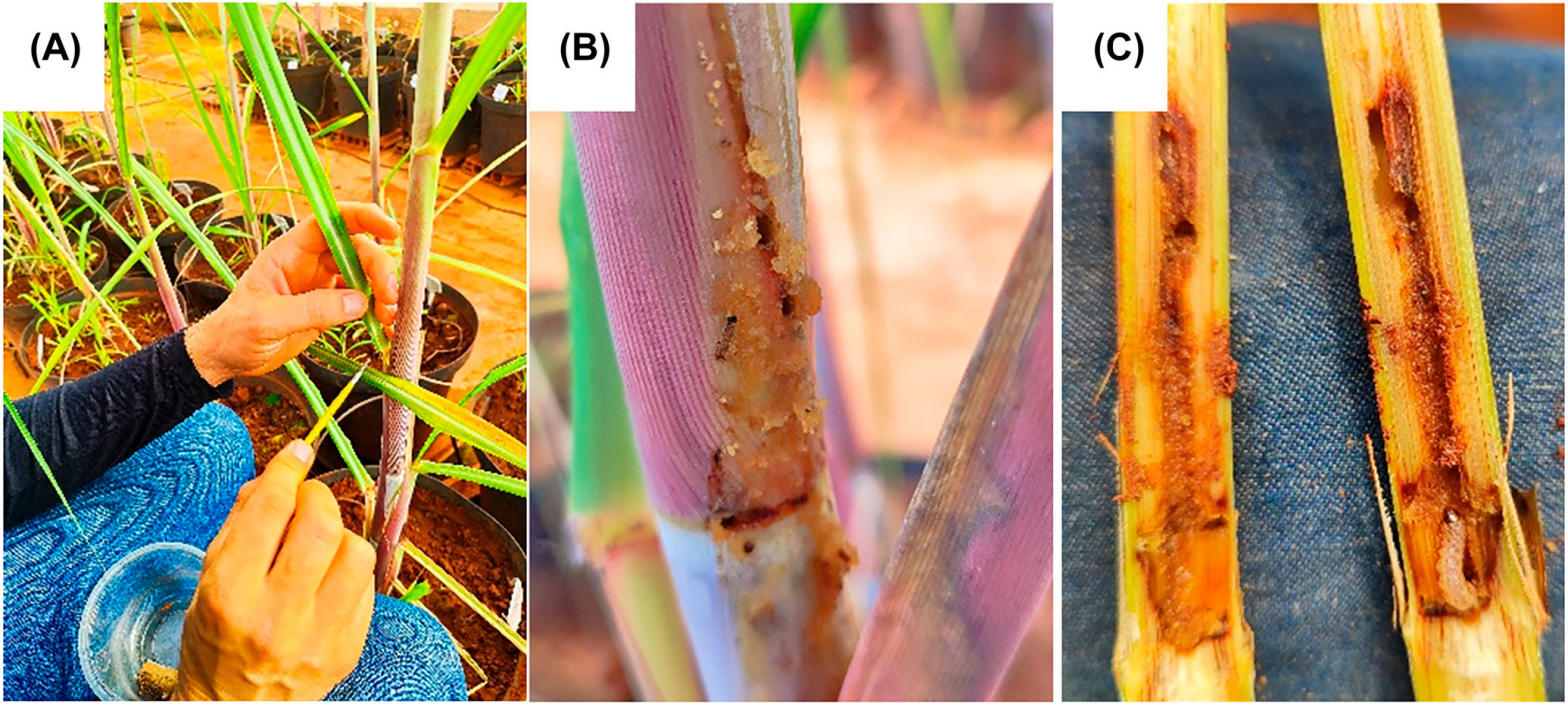
Details of artificial SB infestation: (A) artificial infestation of third‐instar larvae in plants; (B) preliminary inspection of infestations; (C) final inspection to establish the infestation success.

The artificial infestation of SB larvae occurred only when the pots from the water deficit treatments (WSC and WSDS) reached a mass corresponding to the soil water content of 60% WRC. Five third‐instar SB larvae (15 days after emergence of the larvae), supplied by a biofactory of biological control agents, were transferred and positioned with the help of a brush of thin hair close to the sheath of +2 and +3 leaves of the plants (Fig. 1A). We examined the insects for 48 h to confirm the larvae had entered the culm. The survey was based on the observation of the presence of an input hole in the culm (Fig. 1B), and plants were reinfested when necessary.

### Gas exchange measurements

Initial gas exchange parameters were collected 72 h after the artificial infestation of SB larvae and the process was repeated every five days, resulting in four samplings, i.e. 3, 8, 13, and 18 days post‐infestation (DPI). This allowed for analysis during the larval development period of *D. saccharalis*.^26^ In the first evaluation, we randomly selected six pots (replicas) of each treatment from the 20 available and kept them until the end of the experiment. The readings were performed with an IRGA CIRAS‐3 (PP Systems®, Amesbury, MA, USA) between 9:00 a.m. and 11:30 a.m. The reference CO_2_ concentration within the foliar chamber was kept constant at 400 μmol CO_2_ mol^−1^ and light intensity set to 1500 μmol m^−2^ s^−1^. The collection of information consisted of three readings (replicas) at an interval of 10 s in each pot (sampling unit), taken on the +1 leaf (first newly developed leaf, showing the visible auricle), in the median portion. The following parameters were measured: intercellular concentration of CO_2_ (Ci; μmol CO_2_ mol^−1^), stomatal conductivity (gs; mmol H_2_O m^−2^ s), transpiration (E; mmol H_2_O m^−2^ s^−1^), vapor pressure deficit leaf–air (VPD; kPa), photosynthetic water use efficiency (WUE; mmol CO_2_ mol^−1^ H_2_O) and assimilation/respiration (A; μmol CO_2_ m^−2^ s^−1^).

### Foliar spectral measurements

A spectral sensor captured the radiance readings of sugarcane plants subjected to both stresses. The measuring instrument was a portable ASD FieldSpec spectroradiometer, HandHeld UV/VNIR® model, operating in the spectral range between 375 and 1075 nm (Analytical Spectral Devices Inc., Boulder, CO, USA). The measurements were carried out at night to ensure uniformity of lighting. The auxiliary artificial lighting consisted of two reflectors carrying 500 W halogen lamps (Ourolux®, São Paulo, Brazil) positioned at a 30° angle towards the target.^27^ The lamps were turned on 15 min before the readings to stabilize the temperature deviation and ensure the uniformity of the lighting.

Radiometric samples were recorded of the 20 pots by treatment and sugarcane variety directly from the nadir position, with the sensor positioned on a tripod at an approximate height of 20 cm above the +1 leaf of the plants, with restriction of the sensor's target field to 1°. For each sample element, the average of five repetitions of the target radiance reading and the radiance of a reference Lambertian surface a Spectralon® reference diffuse plate (Labsphere Inc., North Sutton, NH, USA) were used in the same lighting and observation conditions to estimate the target's reflectance factor. After the readings, the information was extracted in Viewspec Pro v 6.2 software (Analytical Spectral Devices Inc., Boulder, CO, USA) to obtain the reflected radiance. We then calculated the reflectance using Eqn (1):

(1) $$\;\text{Reflectance}=\frac{R_{\text{target}}}{R_{\text{reference}}}\times K$$

where: *R*_target_ is the radiance reflected by the target, *R*_reference_ is the radiance reflected by the Spectralon® plate, and *K* is the correction factor of the plate.

We converted the radiance values to reflectance, and then used the average of five readings per pot to create a single curve per sample element. We selected readings between 400 and 1000 nm and applied a 5‐point Savitzky–Golay filter to smooth the curves to minimize the noise effect caused by external interference to the target and by the inaccuracies of the instrument.^28^ Readings with values ±5 standard deviations from the overall average were considered ‘outliers’ and discarded from subsequent processing.

### Estimate of SB injury extent impacts on gas exchange traits

At the end of the experiment, all plants were carefully inspected for undue infestations in control plants (no infestation) and the success of infestation in plants intended for this treatment (Fig. 1C). Non‐infested plants were relocated to the control treatment corresponding to the submitted water regime, i.e. uninfected plants from the NSDS treatment were transferred to the NSC. During the inspection of the culms, the length of the SB galleries was also measured to determine the extent of the injury in the sugarcane culms with the help of a measurement tape.

### Data analysis

As the experimental design involved repeated measurements, linear mixed models were adjusted to simultaneously assess the combined effects of the SB and water regime, sugarcane variety, and DPI (fixed effects). The pots (sample units) were considered as a random effect for each of the physiological parameters raised by IRGA. Initially, cases of significant interaction were investigated, comparing the significance of models with interactive and additive factors through a likelihood ratio test. Then, the normality and homogeneity of the residuals were inspected. To determine which group averages were significantly different, gas parameters that had significant effects were subjected to contrast with the *P* value adjusted using the Bejamini–Hockberg method.^29^, ^30^ Further regression models were adjusted considering the data collected at 18 DPI as dependent variables and the length of the SB larva gallery to verify the influence of the injury size and gas exchange parameters.

For the sugarcane foliar reflectance dataset, machine learning classification models were used to categorize the combination of stress classes. The exercise consisted of classifying each sampling date separately, as well as the whole dataset. Modeling was initially implemented by dividing the data randomly for training and testing, considering an 80:20 ratio, respectively. In this sense, each DPI dataset comprised 128 samples for training and 32 for testing or approximately 512 samples for training and 130 samples and testing considering the whole dataset (all DPIs). Multicollinearity, which is prevalent in remote sensing datasets, is known to affect model performance negatively and increase the risk of overfitting.^31^ Therefore, we selected four algorithms, known as multinomial lasso regression, random forest, extreme gradient boosting trees (XGBoost), and sparse partial least squares regression for discriminatory analysis (SPLS‐DA). The chosen algorithms deal with highly correlated predictors internally through feature selection techniques such as recursive feature elimination commonly used in decision trees, coefficient penalization of insignificant variables such as in lasso regression, and a combination of dimensionality reduction and penalization of uninformative variables like in SPLS regression.^32^, ^33^, ^34^ For model fitting process, we adopted the fivefold cross‐validation repeated three times as resampling method. This method allows subdivision of the training data into smaller sets to estimate the test error associated with a given model (model evaluation) and to select the appropriate model's hyperparameters.

Four algorithms were tested to determine the one with the best accuracy and precision of the stressor classes: random decision trees (random forest), XGBoost, multinomial lasso regression and SPLS‐DA.^32^, ^34^, ^35^, ^36^ The models were trained and compared to each other to find out which had the best classification accuracy using the metrics balanced accuracy, F1‐score, precision, and recall for all the subsets of data (Table 3). The algorithm that afforded the best metrics in the training dataset was evaluated in the test data, with the subsequent construction of a confusion matrix.

The analyses were carried out with the packages ‘tidyverse’,^37^ ‘lme4’,^38^ ‘LmerTest’,^39^ ‘DHARMa’^40^ and ‘rstatix’^41^ and the graphs were plotted through the ‘ggpubr’ package.^42^ Classification models were implemented using the ‘Tidymodels’ package.^37^ All analyses were done in R software version 4.3.1.^43^

## RESULTS

### Influence of the combination of stressors on sugarcane gas exchange parameters

**Figure 2:**
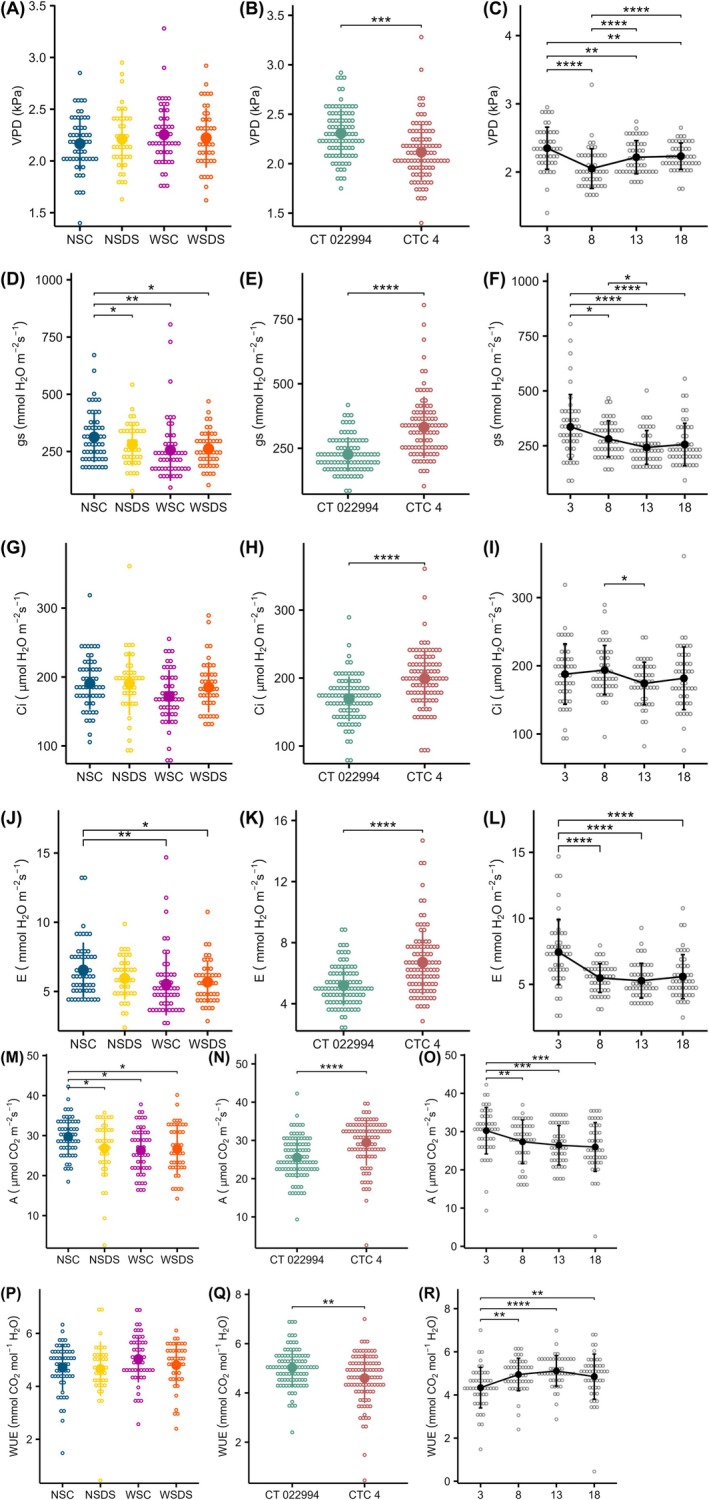
VPD (A–C), gs (D–F), Ci (G–I), E (J–L), A (M–O) and WUE (P–R) of plants subjected to the combination of abiotic and biotic stressors (NSC, no water restriction and no SB infestation; NSDS, no water restriction with SB infestation; WSC, water restriction and no SB infestation; WSDS, water restriction with SB infestation) (A, D, G, J, M, P); sugarcane varieties (CT 022994 and CTC 4) (B, E, H, K, N, Q); and different DPI (3, 8, 13 and 18 DPI) (C, F, I, L, O, R). Filled circles with vertical bars and open circles represent the average value ± standard deviation and raw data, respectively. Brackets delimit significant contrasts. *P < 0.05; **P < 0.01; ***P < 0.001; ****P < 0.0001.

The parameters evaluated showed no significant interaction between sugarcane varieties, treatments (water stress and SB infestation) and DPI (Table S2). As a result, the simple effects of the factors studied were considered. The effects of the stressors did not significantly affect the VPD (*F*_3,43_ = 0.73; *P* = 0.54; $\eta_{p}^{2}=0.05$) (Fig. 2(A)), Ci (*F*_3,43_ = 0.12; *P* = 0.12; $\eta_{p}^{2}=0.12$) (Fig. 2(G)) and WUE (*F*_3,43_ = 0.25; *P* = 0.25; $\eta_{p}^{2}=0.09$) (Fig. 2(P)) in sugarcane leaves (Table S3). Meanwhile, significant changes were observed in the effects of stressors for parameters E (*F*_3,43_ = 5.08; *P* = 0.00042; $\eta_{p}^{2}=0.26$), gs (*F*_3,43_ = 5.70; *P* = 0.0022; $\eta_{p}^{2}=0.28$) and A (*F*_3,43_ = 3.12; *P* = 0.036; $\eta_{p}^{2}=0.18$). The contrasts point out differences primarily between plants not subject to stressors (NSC) and plants in combination with any of the abiotic or biotic stressors (NSDS, WSC and WSDS) (Fig. 2(D),(J),(M)). There was a significant reduction between these parameters with the estimated mean values of 0.17 mmol H_2_O m^−2^ s^−1^, 58.6 mmol m^−2^ s^−1^ and 3.58 μmol CO_2_ m^−2^ s^−1^ for E, gs and A (Table S2).

Differences were observed between the varieties and DPI, regardless of the physiological parameter raised (Table S3). The CTC 4 variety demonstrated higher values among the gas parameters (gs: *F*_1,43_ = 57.97; *P* < 0.0001; $\eta_{p}^{2}=0.57$; Ci: *F*_1,43_ = 22.56; *P* < 0.0001; $\eta_{p}^{2}=0.34$; E: *F*_1,43_ = 36.62; *P* < 0.0001; $\eta_{p}^{2}=0.46$; A: *F*_1,43_ = 17.85; *P* = 0.00012; $\eta_{p}^{2}=0.29$), except VPD and WUE (VPD: *F*_1,43_ = 17.85; *P* = 0.00012; $\eta_{p}^{2}=0.29$; WUE: *F*_1,43_ = 10.04; *P* = 0.0028; $\eta_{p}^{2}=0.19$) (Fig. 2(B),(E),(H),(K),(N),(Q)). Average increases of 16.34% were observed in Ci, 12.82% in E, 6.68% in gs and 13.94% in A, and a decrease of 7.95% and 9.48% in VPD and WUE for variety CTC 4 compared to CT 022994 (Table S2).

**Figure 3:**
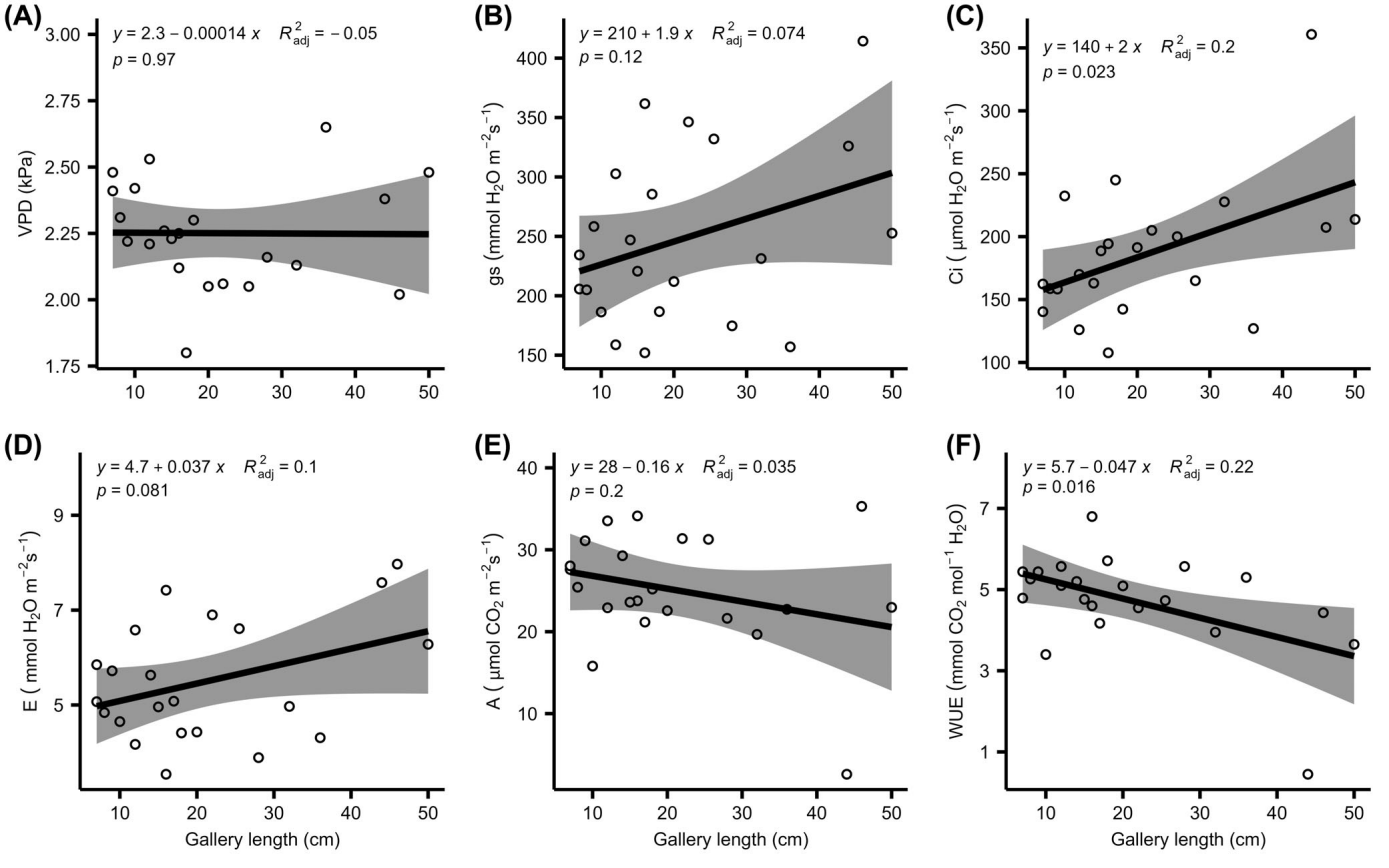
Relationship between the size of the injury of SB and sugarcane gas exchange parameters. The shaded area represents the confidence interval (95%).

The relationship between gallery length and gas exchange response was only significant for Ci and WUE (Fig. 3). For these parameters, there is a twofold increase in Ci and a decrease of 0.047 per centimeter of length of injured tissue by SB larvae for WUE (Fig. 3(C),(F)).

### Water regime and SB combination on sugarcane spectral reflectance

Among the algorithms tested, multinominal regression performed the best over the others. However, their ability to correctly classify treatments was poor, with balanced accuracy values below 0.7 regardless of DPI or complete dataset (Table 2).

**Table 2:** Performance of algorithms tested in the training data. The winning dataset and algorithm are highlighted in bold

Dataset	Algorithm	Balanced accuracy	F1‐score	Precision	Recall
3 DPI	Multinomial regression	0.55 ± 0.01	0.32 ± 0.01	0.32 ± 0.01	0.32 ± 0.01
	Random forest	0.57 ± 0.0	0.32 ± 0.03	0.30 ± 0.03	0.32 ± 0.03
	XGBoost	0.56 ± 0.01	0.31 ± 0.02	0.31 ± 0.02	0.34 ± 0.02
	SPLS‐DA	0.51 ± 0.02	0.26 ± 0.02	0.29 ± 0.02	0.27 ± 0.03
8 DPI	Multinomial regression	0.57 ± 0.01	0.32 ± 0.02	0.33 ± 0.03	0.35 ± 0.02
	Random forest	0.53 ± 0.01	0.27 ± 0.02	0.32 ± 0.03	0.29 ± 0.02
	XGBoost	0.50 ± 0.00	0.45 ± 0.03	0.30 ± 0.02	0.25 ± 0.00
	SPLS‐DA	0.52 ± 0.01	0.26 ± 0.02	0.30 ± 0.03	0.29 ± 0.02
13 DPI	**Multinomial regression**	**0.64 ± 0.02**	**0.44 ± 0.02**	**0.48 ± 0.03**	**0.46 ± 0.02**
	Random forest	0.63 ± 0.02	0.43 ± 0.02	0.45 ± 0.02	0.45 ± 0.02
	XGBoost	0.63 ± 0.02	0.43 ± 0.03	0.45 ± 0.03	0.44 ± 0.03
	SPLS‐DA	0.64 ± 0.02	0.42 ± 0.03	0.45 ± 0.04	0.45 ± 0.03
18 DPI	Multinomial regression	0.57 ± 0.02	0.32 ± 0.02	0.35 ± 0.03	0.35 ± 0.02
	Random forest	0.55 ± 0.01	0.29 ± 0.01	0.33 ± 0.02	0.33 ± 0.02
	XGBoost	0.54 ± 0.01	0.28 ± 0.02	0.31 ± 0.03	0.31 ± 0.02
	SPLS‐DA	0.57 ± 0.02	0.33 ± 0.03	0.33 ± 0.03	0.36 ± 0.02
All DPI	Multinomial regression	0.55 ± 0.01	0.32 ± 0.01	0.32 ± 0.01	0.32 ± 0.01
	Random forest	0.58 ± 0.01	0.35 ± 0.01	0.36 ± 0.01	0.36 ± 0.01
	XGBoost	0.56 ± 0.01	0.33 ± 0.01	0.34 ± 0.01	0.34 ± 0.01
	SPLS‐DA	0.53 ± 0.01	0.26 ± 0.01	0.31 ± 0.02	0.29 ± 0.01

DPI, days after infestation.

The 13 DPI dataset presented the best performance with values above 0.60 for balanced accuracy and F1‐score, precision, and recall above 0.4 (Table 2).

From the 13 DPI dataset, the multinominal regression obtained a balanced accuracy of 0.72 in the test data (Table 3). The confusion matrix shows that there were problems when separating classes referring to treatments that share the same water regime (Table 3).

**Table 3:** Metrics and confusion matrix of the winning algorithm at 13 DPI

Dataset	Algorithm	Balanced accuracy	F1‐score	Precision	Recall
13 DPI	Multinomial regression	0.72	0.57	0.58	0.58
Penalization: 1.51 × 10^−10^					

Confusion matrix					
Observed					
		NSC	NSDS	WSC	WSDS
Predicted	NSC	8	1	1	1
	NSDS	2	3	0	1
	WSC	1	1	4	2
	WSDS	0	1	2	4

DPI, days after infestation; NSC, no water stressed, no SB infestation; NSDS, no water stressed, SB infested; WSC, water stressed, no SB infestation; WSDS, water stressed, SB infested.

## DISCUSSION

In our study model, we sought to investigate the relationship between injury caused by the SB, a culm‐boring phytophagous insect, and controlled water stress (60% WRC), isolated or combined, and the gas exchange parameters of the leaves in two sugarcane varieties. Our hypothesis was that SB larva attack could interfere with the transport of water and nutrients by destroying the culm conductive vessels. Such an effect could mimic symptoms similar to water deficiency, but it would be possible to differentiate sugarcane plant responses between the two stressors. In fact, it has been revealed that there is an interference of SB attack in the physiology of the sugarcane, although it has not been possible to differentiate its effects from those caused by the established water stress level. We showed that both stresses, isolated or combined, interfere with some of the gas exchange and spectral reflectance parameters evaluated, regardless of the post‐introduction period of stress and sugarcane variety. Furthermore, the responses between the varieties differ from one another.

The parameters with significant differences due to stressors were E, gs, and A. These results agree with those of several other studies on the physiological response of plants to water deficits.^4^, ^12^, ^44^, ^45^ Stomatal closure, or reduced stomatal conductivity, typically accompanies the reduction of liquid photosynthesis. This, in turn, restricts CO_2_ entry into the stomata, resulting in a reduced transpiration coefficient in drier conditions.^2^, ^3^, ^14^

However, the effects of phytophagous insect attacks on the physiology of cultivated plants may vary.^46^, ^47^ Several reports highlight no significant effect on the photosynthesis rate, stomatal conductivity, Ci, and evaporation, especially in periods close to the introduction of the stressor.^10^, ^45^, ^46^, ^47^ In our case, the reduction of E, gs, and A parameters was detected 72 h after plant infestation and remained so throughout the evaluation period, compared with plants that did not experience any kind of stress. Our findings suggest that the effects of herbivory induced by *D. sacharalis* larvae appear to be similar to those of the specified water stress level. Results of a field study also showed negative impacts between photosynthetic rates of uninfested plants and plants infested with SB, especially in the drier seasons of the year.^9^ However, further in‐depth research is needed to verify whether such responses would not alter in plants subjected to more pronounced water stress levels and the injury imposed by SB larvae.

The spectral response of the sugarcane plants was similar among the treatments, since classification metrics were low regardless of the period analyzed. The confusion matrix emphasizes that incorrect classifications primarily involved treatments with similar water regimes, suggesting that the imposed water stress had a more significant impact on the sugarcane's spectral reflectance than SB. In this way, detecting SB attacks through remote sensing may be challenging. Such adversities are intensified in field conditions since in the sugarcane agroecosystem, there are other stress agents that interact with sugarcane and their effects are similar to each other.^19^, ^48^ These findings suggest that the use of remote sensing with the objective of SB monitoring may not be ideal, as the plant would change its spectral profile after larvae bore into the culm. Consequently, the detection of the SB would occur after the damage occurred and at a time of difficult control since the larvae would be protected inside the culm.^49^

When considering the same injury guild, our results show similar effects to those found by the attack of the European maize borer, *Ostrinia nubilalis* (Hubner 1796) (Lepidoptera: Pyralidae), on corn physiology.^44^ The attack of European corn borer had similarities with the effects of water stress on the plant (through a reduction in A, gs, and Ci and an increase in leaf temperature), as observed by Godfrey *et*. *al*.^44^ Similarly, another culm borer, the wheat stem sawfly *Cephus cinctus* Norton, 1872, reduced the liquid photosynthesis of wheat plants regardless of the established water regime.^50^ But, unlike maize and wheat, sugarcane infestations with *D. saccharalis* are associated with fungal infections from *Colletotrichum* spp. and *Fusarium* spp., causing red rot symptoms.^15^ These symptoms are associated with the biochemical defenses of plants through the production of phenols to combat such etiological agents.^15^, ^51^ As a result, the presence of these fungi can have a negative impact on sugarcane's physiology. However, separating and quantifying these effects from those caused by the SB is a difficult task since the fungi are always found associated with insect injury.^52^

The difference in response between varieties for all parameters was an expected result since they were different genotypes. Interestingly, the variety CT 022994, recognized for its water stress tolerance,^53^ showed lower values of the gas parameters, except for VPD and WUE. This observation may be associated with differences in the developmental cycle and environmental production requirements among varieties. As recommended by the Centro de Tecnologia Canavieira, the CTC 4 variety has a medium cycle and can be allocated in more restrictive environments.^53^ In contrast, the variety CT 022994 has a late cycle and is recommended for more productive sites. In an ecological context, the CTC 4 variety would have less time for its development and, consequently, its ripening. Therefore, this genotype would need to produce and accumulate more photoassimilates to compensate for the time lag to maturity, in contrast to CT 022994.

When considering the extent of the SB injury in the expression of stress in sugarcane plants, it was intuitively expected that there would be a positive relationship between the length of the gallery inflicted by larvae and the plant's response to stress. However, this hypothesis has not been proved, which has also not been confirmed in studies on the injury of *D. saccharalis* in rice culture.^54^ Apparently, SB does not destroy all the conductive vessels of the culm, and there is possibly a tolerance or compensation mechanism of the plant. In rice, compensation occurs by issuing new tillers to plants injured in the stem or by increasing the size of the panicles when the injury is observed in the leaves and leaf sheath.^54^ In our case, there are indications for the assumption of compensation, as it was observed that Ci and WUE had significant relationships with the size of the injury; that is, in some way, the plant would increase its internal concentration of CO_2_ and would lose more water to keep a level of net photosynthesis that would compensate for the stress imposed.

This finding would have relevant ecological significance because if the insect severely affects its host, potentially leading to its death, it could compromise its own development. Conversely, maize plants infested with SB showed contradictory results to those found in this study, in which higher WUE and lower E were observed.^55^ Although the three crops share the same family (Poaceae), maize does not have tillering ability like rice and sugarcane, and it is the crop that has undergone the greatest selection pressure through genetic breeding. Consequently, maize may have lost characteristics that confer tolerance to stressors.^56^ Furthermore, our findings suggest that the economic losses associated with SB injury may often be exacerbated by secondary infections caused by fungi of the red rot complex. Although red rot disease can occur independently of SB infestation, field observations frequently report the co‐occurrence of both, indicating that SB injury may facilitate fungal entry in certain conditions.^57^ This highlights the potential role of SB as a facilitator of fungal infection, rather than attributing economic losses solely to its direct damage.^52^

In summary, our results show that physiological and spectral responses to moderate water restriction are difficult to distinguish from each other. Understanding how sugarcane tolerates damage from the SB and other stressors is crucial to improve the economic thresholds for managing this pest. If variations are observed in the sugarcane tolerance imposed by environmental conditions, such levels can be changed. Conversely, if sugarcane tolerance can be improved by environmental conditions, there may be no need for control. In this sense, our work brings evidence that moderate water stress does not alter the sugarcane tolerance to the damage caused by SB since plants attacked by the pest had a reduction in liquid photosynthesis similar to plants subjected to both stresses and water deficit alone.

## AUTHOR CONTRIBUTIONS

João RS Soares: investigation, formal analysis, writing – original draft; Vinicius Cesarin: investigation, data curation; Claudiane Martins da Rocha: investigation, methodology; Júlia Karoline Mercês: investigation, data curation; Fernanda Sayuri Yoshino Watanabe: visualization, writing – review & editing; Nilton Nobuhiro Imai: visualization, supervision, writing – review & editing; Antonio Maria Garcia Tommaselli: visualization, writing – review & editing, funding acquisition; Priscila Lupino Gratão: visualization, writing – review & editing; Odair Aparecido Fernandes: supervision, writing – review & editing, funding, project administration.

## CONFLICT OF INTEREST

The authors declare that they have no known competing financial interests or personal relationships that could have appeared to influence the work reported in this paper.

## FUNDING INFORMATION

This work was supported by Coordenação de Aperfeiçoamento de Pessoal de Nível Superior – Finance code 001, the High‐resolution remote sensing for digital agriculture project (FAPESP; grant no. 2021/06029‐7) and CEPENFITO – Engineering Research Center for Sugarcane Plant Health (FAPESP; grant no. 2017/25258‐1). JRSS received a scholarship through FAPESP (grant no. 2021/09645‐0).

## Supporting information


**TABLE S1.**Mixed Linear Models (MLM) candidates tested by likelihood ratio test for gas exchange parameters. Each candidate model is compared with the simplest model (additive) and is selected only if *P* < 0.05.
**TABLE S2.** Estimate of gas exchange parameters for the selected mixed linear model.
**TABLE S3.** ANOVA table from Mixed Linear Models (MLM) considering the effects of sugarcane varieties, biotic and abiotic stressors and time after sugarcane borer infestation (DPI) on gas exchange parameters.

## Data Availability

The data that support the findings of this study are available on request from the corresponding author. The data are not publicly available due to privacy or ethical restrictions.

## References

[1] Ramegowda V , Senthil A and Senthil‐Kumar M , Stress combinations and their interactions in crop plants. Plant Phys Rep 29:1–5 (2024).

[2] Nawaz M , Sun J , Shabbir S , Khattak WA , Ren G , Nie X *et al*., A review of plants strategies to resist biotic and abiotic environmental stressors. Sci Total Environ 900:165832 (2023). 37524179 10.1016/j.scitotenv.2023.165832

[3] Azevedo RA , Carvalho RF , Cia MC and Gratão PL , Sugarcane under pressure: an overview of biochemical and physiological studies of abiotic stress. Trop Plant Bio 4:42–51 (2011).

[4] Pascali M , Vergine M , Sabella E , Aprile A , Nutricati E , Nicolì F *et al*., Molecular effects of *Xylella fastidiosa* and drought combined stress in olive trees. Plants 8:437 (2019). 31652681 10.3390/plants8110437 PMC6918294

[5] Zhong J , Zhang J , Zhang Y , Ge Y , He W , Liang C *et al*., Heat stress reprograms herbivory‐induced defense responses in potato plants. BMC Plant Biol 24:1–18 (2024). 39014327 10.1186/s12870-024-05404-x PMC11253553

[6] Pandey P , Irulappan V , Bagavathiannan MV and Senthil‐Kumar M , Impact of combined abiotic and biotic stresses on plant growth and avenues for crop improvement by exploiting physio‐morphological traits. Front Plant Sci 8:237767 (2017). 10.3389/fpls.2017.00537 PMC5394115 28458674

[7] Ramegowda V , Da CMVJ , Harihar S , Karaba NN and Sreeman SM , Abiotic and biotic stress interactions in plants: a cross‐tolerance perspective, in Priming‐Mediated Stress and Cross‐Stress Tolerance in Crop Plants, ed. by Hossain MA , Liu F , Burritt DJ , Fujita M and Huang B . Academic Press, London, UK, pp. 267–302 (2020).

[8] Haile FJ , Drought stress, insects, and yield loss, in Biotic Stress and Yield Loss, 1st edn, ed. by Peterson RKD and Higley LG . CRC Press, Boca Raton, FL, pp. 117–133 (2000).

[9] Rossato JAS , Madaleno LL , Mutton MJR , Higley LG and Fernandes OA , Photosynthesis, yield and raw material quality of sugarcane injured by multiple pests. PeerJ 2019:e6166 (2019). 10.7717/peerj.6166 PMC6340350 30687588

[10] Savi T , García González A , Herrera JC and Forneck A , Gas exchange, biomass and non‐structural carbohydrates dynamics in vines under combined drought and biotic stress. BMC Plant Biol 19:1–11 (2019). 31533621 10.1186/s12870-019-2017-2 PMC6749654

[11] de Porto NA , Roque JV , Wartha CA , Cardoso W , Peternelli LA , Barbosa MHP *et al*., Early prediction of sugarcane genotypes susceptible and resistant to *Diatraea saccharalis* using spectroscopies and classification techniques. Spectrochim Acta A 218:69–75 (2019). 10.1016/j.saa.2019.03.114 30954799

[12] Quandahor P , Lin C , Gou Y , Coulter JA and Liu C , Leaf morphological and biochemical responses of three potato (*Solanum tuberosum* L.) cultivars to drought stress and aphid (*Myzus persicae* Sulzer) infestation. Insects 10:435 (2019). 31817160 10.3390/insects10120435 PMC6956135

[13] Lakshmanan P and Robinson N , Stress physiology: abiotic stresses, in Sugarcane: Phsiology, Biochemistry and Functional Biology, ed. by Moore PH and Botha FC , John Wiley & Sons, Chichester, UK, pp. 411–434 (2013).

[14] Siddique Z , Jan S , Imadi SR , Gul A and Ahmad P , Drought stress and photosynthesis in plants, in Water Stress and Crop Plants: A Sustainable Approach, ed. by Ahmad P . John Wiley & Sons, Chichester, UK, pp. 1–11 (2016).

[15] Sathyabhama M , Viswanathan R , Nandakumar M , Malathi P and Ramesh SA , Understanding sugarcane defence responses during the initial phase of *Colletotrichum falcatum* pathogenesis by suppression subtractive hybridization (SSH). Physiol Mol Plant Pathol 91:131–140 (2015).

[16] Nilsson H , Remote sensing and image analysis in plant pathology. Ann Rev Phytopath 33:489–528 (1995). 10.1146/annurev.py.33.090195.002421 18999971

[17] Ennouri K and Kallel A , Remote sensing: an advanced technique for crop condition assessment. Math Problems Eng 2019:1–8 (2019).

[18] Jensen JR , Remote Sensing of the Environment: An Earth Resource Perspective, 2nd edn. Pearson Prentice Hall, Upper Saddle River, NJ (2007).

[19] Zhang J , Huang Y , Pu R , Gonzalez‐Moreno P , Yuan L , Wu K *et al*., Monitoring plant diseases and pests through remote sensing technology: a review. Comp Elec Agri 165:104943 (2019).

[20] Mahanta DK , Bhoi TK , Komal J , Samal I and Mastinu A , Spatial, spectral and temporal insights: harnessing high‐resolution satellite remote sensing and artificial intelligence for early monitoring of wood boring pests in forests. Plant Stress 11:100381 (2024).

[21] Peterson RKD , Higley LG and Pedigo LP , Whatever happened to IPM? Am Entomol 64:146–150 (2018).

[22] Sandhu HS and Cherry RH , Sugarcane borer, *Diatraea saccharalis* (F.) (Lepidoptera: Crambidae), injury and survival in energy cane versus sugarcane. Sugar Tech 20:558–565 (2018).

[23] Rossato Junior JAS , Costa GH , Madaleno LL , Mutton MJ , Higley LG and Fernandes OA , Characterization and impact of the sugarcane borer on sugarcane yield and quality. Agro J 105:643–648 (2013).

[24] Landell MGA , Campana MP , Figueiredo P , Xavier MA and Anjos IA , Sistema de multiplicação de cana‐de‐açúcar com uso de mudas pré‐brotadas (MPB), oriundas de gemas individualizadas. Instituto Agronômico de Campinas, Campinas, Brazil, pp. 1–16 (2013).

[25] Braga Junior RLC , Landell MG , Xavier MA , Kanthack RAD , Silva DN , Bidóia MAP *et al*., Censo varietal IAC de cana‐de‐açúcar no Brasil‐Safra 2022/23 Campinas, SP; p. 63 2023. Available: https://www.iac.sp.gov.br/media/publicacoes/iacbt235.pdf [4 August 2024].

[26] Tomaz AC , Coutinho AE , Soares BO , Peternelli LA , Pereira EJG and Barbosa MHP , Assessing resistance of sugarcane varieties to sugarcane borer *Diatraea saccharalis* Fab. (Lepidoptera Crambidae). Bull Ento Res 108:547–555 (2018). 10.1017/S0007485317001183 29198198

[27] Vohland M , Besold J , Hill J and Fründ HC , Comparing different multivariate calibration methods for the determination of soil organic carbon pools with visible to near infrared spectroscopy. Geoderma 166:198–205 (2011).

[28] Savitzky A and Golay MJ , Smoothing and differentiation of data by simplified least squares procedures. Anal Chem 36:1627–1639 (1964).

[29] Benjamini Y and Hochberg Y , Controlling the false discovery rate: a practical and powerful approach to multiple testing. J R Stat Soc Ser B 57:289–300 (1995).

[30] Waite TA and Campbell LG , Controlling the false discovery rate and increasing statistical power in ecological studies. Écoscience 13:439–442 (2006).

[31] Chan JY‐L , Leow SMH , Bea KT , Cheng WK , Phoong SW , Hong Z‐W *et al*., Mitigating the multicollinearity problem and its machine learning approach: a review. Mathematics 10:1283 (2022).

[32] Chen T and Guestrin C , XGBoost: a scalable tree boosting system, in Proceedings of the ACM SIGKDD International Conference on Knowledge Discovery and Data Mining. Association for Computing Machinery, New York, USA, pp. 785–794 (2016).

[33] Hengl T , Nussbaum M , Wright MN , Heuvelink GBM and Gräler B , Random forest as a generic framework for predictive modeling of spatial and spatio‐temporal variables. PeerJ 6:5518 (2018). 10.7717/peerj.5518 PMC6119462 30186691

[34] Chun H and Keleş S , Sparse partial least squares regression for simultaneous dimension reduction and variable selection. J R Stat Soc Ser B 72:3–25 (2010). 10.1111/j.1467-9868.2009.00723.x PMC2810828 20107611

[35] Belgiu M and Drăgu L , Random forest in remote sensing: a review of applications and future directions. ISPRS J Photogramm Remote Sens 114:24–31 (2016).

[36] Nibbering D and Hastie TJ , Multiclass‐penalized logistic regression. Comput Statist Data Anal 169:107414 (2022).

[37] Kuhn M and Wickham H , Tidymodels: a collection of packages for modeling and machine learning using tidyverse principles (2020).

[38] Bates D , Mächler M , Bolker B and Walker S , Fitting linear mixed‐effects models using lme4. J Stat Softw 67:1–48 (2015).

[39] Kuznetsova A , Brockhoff PB and Christensen RHB , lmerTest package: tests in linear mixed effects models. J Stat Softw 82:1–16 (2017).

[40] Hartig F , DHARMa: residual diagnostics for hierarchical (multi‐level/mixed) regression models. R package version (2022).

[41] Kassambara A , rstatix: pipe‐friendly framework for basic statistical tests (2023).

[42] Kassambara A , ggpubr: ‘ggplot2’ based publication ready plots (2023).

[43] R Core Team , R: a language and environment for statistical computing. Vienna, Austria: R Foundation for Statistical Computing 2023. Available: https://www.r-project.org/ [15 January 2024].

[44] Godfrey LD , Holtzer TO and Norman JM , Effects of European corn borer (Lepidoptera: Pyralidae) tunneling and drought stress on field corn gas exchange parameters. J Eco Ento 84:1370–1380 (1991).

[45] Heimoana SC , Wilson LJ , Constable GA and George DL , Do phloem feeders affect gas exchange? A case study of *Aphis gossypii* (Glover) on cotton. Crop Sci 63:912–920 (2022).

[46] Welter SC , Arthropod impact on plant gas exchange, in Insect‐Plant Interactions, 1st edn, ed. by Bernays EA . CRC Press, Boca Raton, FL, pp. 135–164 (1989).

[47] Retuerto R , Fernanez‐Lema B , Roiloa R and Obeso JR , Increased photosynthetic performance in holly trees infested by scale insects. Func Ecol 18:664–669 (2004).

[48] Yuan L , Bao Z , Zhang H , Zhang Y and Liang X , Habitat monitoring to evaluate crop disease and pest distributions based on multi‐source satellite remote sensing imagery. Optik (Stuttg) 145:66–73 (2017).

[49] Li AM , Chen ZL , Liao F , Zhao Y , Qin CX , Wang M *et al*., Sugarcane borers: species, distribution, damage and management options. J Pest Sci 97:1171–1201 (2024).

[50] Macedo TB , Weaver DK and Peterson RKD , Photosynthesis in wheat at the grain filling stage is altered by larval wheat stem sawfly (Hymenoptera: Cephidae) injury and reduced water availability. J Entomol Sci 42:228–238 (2007).

[51] Ogunwolu EO , Reagan TE , Flynn JL and Hensley SD , Effects of *Diatraea saccharalis* (F.) (Lepidoptera: Pyralidae) damage and stalk rot fungi on sugarcane yield in Louisiana. Crop Prot 10:57–61 (1991).

[52] Franco FP , Túler AC , Gallan DZ , Gonçalves FG , Favaris AP , Peñaflor MFGV *et al*., *Colletotrichum falcatum* modulates the olfactory behavior of the sugarcane borer, favoring pathogen infection. FEMS Microbiol Ecol 98:1–8 (2022). 10.1093/femsec/fiac035 35333339

[53] CTC. Variedades CTC , CTC‐Centro de Tecnologia Canavieira (2024). Available: https://ctc.com.br/produtos/ [23 January 2024].

[54] Lv J , Wilson LT and Longnecker MT , Tolerance and compensatory response of rice to sugarcane borer (Lepidoptera: Crambidae) injury. Environ Ento 37:796–807 (2008). 10.1603/0046-225x(2008)37[796:tacror]2.0.co;2 18559187

[55] Castro MGB , Respostas fisiológicas e espectrais de plantas de milho infestadas por *Diatraea saccharalis* (Fabricius, 1794) (Lepidoptera: Crambidae) e *Spodoptera frugiperda* (J. E. Smith, 1797) (Lepidoptera: Noctuidae). [41 f]: Dissertação (Mestrado em Agronomia‐Entomologia Agrícola) Unesp‐Jaboticabal (2023).

[56] Marone D , Russo MA , Mores A , Ficco DBM , Laidò G , Mastrangelo AM *et al*., Importance of landraces in cereal breeding for stress tolerance. Plants 10:1267 (2021). 34206299 10.3390/plants10071267 PMC8309184

[57] Costa MM , Silva BAAS , Moreira GM and Pfenning LH , *Colletotrichum falcatum* and *Fusarium* species induce symptoms of red rot in sugarcane in Brazil. Plant Pathol 70:1807–1818 (2021).

