# Supplementary material for: An investigation into the interaction between water deficit and injury of sugarcane borer (Lepidoptera: Crambidae) in the gas exchange parameters and spectral reflectance of sugarcane
Source: J Sci Food Agric. 2025 Jul 31;105(15):8551–60. doi: 10.1002/jsfa.70093 (PMC12595397; doi:10.1002/jsfa.70093)
Supplement: Supplementary file 1 — TABLE S1.Mixed Linear Models (MLM) candidates tested by likelihood ratio test for gas exchange parameters. Each candidate model is compared with the simplest model (additive) and is selected only if P < 0.05. TABLE S2. Estimate of gas exchange parameters for the selected mixed linear model. TABLE S3. ANOVA table from Mixed Linear Models (MLM) considering the effects of sugarcane varieties, biotic and abiotic stressors and time after sugarcane borer infestation (DPI) on gas exchange parameters. [file JSFA-105-8551-s001.docx]

**Supplementary Table 1.** Mixed Linear Models (MLM) candidates tested by likelihood ratio test for gas exchange parameters. Each candidate model is compared with the simplest model (additive) and is selected only if p < 0.05

| Parameter | Candidate model* | No. parameterers | AIC | BIC | χ2 | df | P value |
| --- | --- | --- | --- | --- | --- | --- | --- |
| Ci | Ci= β0 + β1VARIETYijk * β2STRESSORSijk * β3DPIijk + ui + εijk | 34 | 1943.8 | 054.6 | 24.19 | 15 | 0.06188 |
|  | Ci= β0 + β1VARIETYijk * β2STRESSORSijk + β3DPIijk + ui + εijk | 13 | 1936.6 | 1978.9 | 4.49 | 3 | 0.21290 |
|  | Ci= β0 + β1VARIETYijk + β2STRESSORSijk * β3DPIijk + ui + εijk | 19 | 1938.0 | 1999.9 | 10.53 | 6 | 0.10387 |
|  | Ci= β0 + β1VARIETYijk + β2STRESSORSijk + β3DPIijk + ui + εijk | 10 | 1935.1 | 1967.7 |  |  |  |
| E | E= β0 + β1VARIETYijk * β2STRESSORSijk * β3DPIijk + ui + εijk | 34 | 9.06 | 119.81 | 29.47 | 15 | 0.17138 |
|  | E= β0 + β1VARIETYijk * β2STRESSORSijk + β3DPIijk + ui + εijk | 13 | -3.66 | 38.68 | 6.50 | 3 | 0.15917 |
|  | E= β0 + β1VARIETYijk + β2STRESSORSijk * β3DPIijk + ui + εijk | 19 | -0.93 | 60.96 | 9.27 | 6 | 0.15917 |
|  | E= β0 + β1VARIETYijk + β2STRESSORSijk + β3DPIijk + ui + εijk | 10 | -3.17 | 29.41 |  |  |  |
| gs | gs= β0 + β1VARIETYijk * β2STRESSORSijk * β3DPIijk + ui + εijk | 34 | 83.83 | 194.59 | 21.97 | 15 | 0.10856 |
|  | gs= β0 + β1VARIETYijk * β2STRESSORSijk + β3DPIijk + ui + εijk | 13 | 74.62 | 116.96 | 9.16 | 3 | 0.06274 |
|  | gs= β0 + β1VARIETYijk + β2STRESSORSijk * β3DPIijk + ui + εijk | 19 | 75.81 | 37.81 | 10.81 | 6 | 0.09443 |
|  | gs= β0 + β1VARIETYijk + β2STRESSORSijk + β3DPIijk + ui + εijk | 10 | 77.78 | 110.35 |  |  |  |

*u_i_ ∼ N(0, $\sigma_{u}^{2}$), ε_ijk_ ∼ N(0, $\sigma^{2}$)

**Supplementary Table 1 (continuation)**

| Parameter | Candidate model* | No. parameterers | AIC | BIC | χ2 | df | P value |
| --- | --- | --- | --- | --- | --- | --- | --- |
| VPD | VPD= β_0_ + β_1_VARIETY_ijk_ * β_2_STRESSORS_ijk_ * β3DPI_ijk_ + u_i_ + ε_ijk_ | 34 | 14.42 | 125.17 | 6.62 | 15 | 0.9674 |
|  | VPD= β_0_ + β_1_VARIETY_ijk_ * β_2_STRESSORS_ijk_ + β3DPI_ijk_ + u_i_ + ε_ijk_ | 13 | -10.42 | 31.92 | 1.65 | 3 | 0.6486 |
|  | VPD= β_0_ + β_1_VARIETY_ijk_ + β_2_STRESSORS_ijk_ * β3DPI_ijk_ + u_i_ + ε_ijk_ | 19 | -8.97 | 52.93 | 10.54 | 6 | 0.1036 |
|  | **VPD= β_0_ + β_1_VARIETY_ijk_ + β_2_STRESSORS_ijk_ + β3DPI_ijk_ + u_i_ + ε_ijk_** | **10** | **-14.78** | **17.80** |  |  |  |
| WUE | WUE= β_0_ + β_1_VARIETY_ijk_ * β_2_STRESSORS_ijk_ * β3DPI_ijk_ + u_i_ + ε_ijk_ | 34 | 506.40 | 617.15 | 21.96 | 15 | 0.1088 |
|  | WUE= β_0_ + β_1_VARIETY_ijk_ * β_2_STRESSORS_ijk_ + β3DPI_ijk_ + u_i_ + ε_ijk_ | 13 | 491.57 | 533.92 | 5.58 | 3 | 0.1339 |
|  | WUE= β_0_ + β_1_VARIETY_ijk_ + β_2_STRESSORS_ijk_ * β3DPI_ijk_ + u_i_ + ε_ijk_ | 19 | 498.36 | 560.25 | 5.21 | 6 | 0.5169 |
|  | **WUE= β_0_ + β_1_VARIETY_ijk_ + β_2_STRESSORS_ijk_ + β3DPI_ijk_ + u_i_ + ε_ijk_** | **10** | **491.15** | **523.73** |  |  |  |
| A | A= β_0_ + β_1_VARIETY_ijk_ * β_2_STRESSORS_ijk_ * β3DPI_ijk_ + u_i_ + ε_ijk_ | 34 | 1227.1 | 1337.8 | 10.89 | 15 | 0.76 |
|  | A= β_0_ + β_1_VARIETY_ijk_ * β_2_STRESSORS_ijk_ + β3DPI_ijk_ + u_i_ + ε_ijk_ | 13 | 1199.3 | 1241.7 | 2.53 | 3 | 0.4692 |
|  | A= β_0_ + β_1_VARIETY_ijk_ + β_2_STRESSORS_ijk_ * β3DPI_ijk_ + u_i_ + ε_ijk_ | 19 | 1208.0 | 1269.9 | 3.37 | 6 | 0.7610 |
|  | **A= β_0_ + β_1_VARIETY_ijk_ + β_2_STRESSORS_ijk_ + β3DPI_ijk_ + u_i_ + ε_ijk_** | **10** | **1195.9** | **1228.5** |  |  |  |

*u_i_ ∼ N(0, $\sigma_{u}^{2}$), ε_ijk_ ∼ N(0, $\sigma^{2}$)

**Supplementary Table 2.** Estimate of gas exchange parameters for the selected mixed linear model

| Parameter | Term | Estimate | SD | df | t value | P value |
| --- | --- | --- | --- | --- | --- | --- |
| Ci | **Intercept** | **181.30** | **7.63** | **86.068** | **23.76** | **2.00 10^-16^** |
|  | **Variety (CTC 4)** | **29.63** | **6.24** | **43** | **4.75** | **2.29 10^-5^** |
|  | NSDS | -4.68 | 8.93 | 43 | -0.52 | 0.6029 |
|  | **WSC** | **-19.75** | **8.25** | **43** | **-2.39** | **0.0211** |
|  | WSDS | -8.88 | 8.66 | 43 | -1.03 | 0.3108 |
|  | 8 DPI | 5.97 | 6.99 | 141 | 0.85 | 0.3947 |
|  | 13 DPI | -13.63 | 6.99 | 141 | -195 | 0.0533 |
|  | 18 DPI | -6.09 | 6.99 | 141 | -0.87 | 0.3855 |
| E | **Intercept** | **1.95** | **0.05** | **80.85** | **38.59** | **2.00 10^-16^** |
|  | **Variety (CTC 4)** | **0.25** | **0.042** | **43** | **6.05** | **2.00 10^-16^** |
|  | **NSDS** | **-0.14** | **0.060** | **43** | **-2.26** | **0.0289** |
|  | **WSC** | **-0.20** | **0.056** | **43** | **-3.67** | **0.00067** |
|  | **WSDS** | **-0.17** | **0.058** | **43** | **-2.84** | **0.0069** |
|  | **8 DPI** | **-0.27** | **0.044** | **141** | **-6.16** | **7.32 10^-9^** |
|  | **13 DPI** | **-0.32** | **0.044** | **141** | **-7.18** | **3.66 10^-11^** |
|  | **18 DPI** | **-0.27** | **0.044** | **141** | **-6.18** | **6.36 10^-9^** |
| gs | **Intercept** | **5.69** | **0.061** | **84.59** | **93.24** | **2.00 10^-16^** |
|  | **Variety (CTC 4)** | **0.38** | **0.050** | **43** | **7.61** | **1.68 10^-9^** |
|  | **NSDS** | **-0.18** | **0.071** | **43** | **-2.50** | **0.0161** |
|  | **WSC** | **-0.25** | **0.066** | **43** | **-3.83** | **0.00041** |
|  | **WSDS** | **-0.21** | **0.070** | **43** | **-3.07** | **0.0036** |
|  | **8 DPI** | **-0.13** | **0.055** | **141** | **-2.32** | **0.021** |
|  | **13 DPI** | **-0.28** | **0.055** | **141** | **-5.02** | **1.51 10^-6^** |
|  | **18 DPI** | **-0.24** | **0.055** | **141** | **-4.36** | **2.46 10^-5^** |

**Supplementary Table 2 (continuation)**

| Parameter | Term | Estimate | SD | df | t value | | P value |
| --- | --- | --- | --- | --- | --- | --- | --- |
| VPD | **Intercept** | **2.39** | **0.059** | **60.80** | | **40.26** | **2.00 10^-16^** |
|  | **Variety (CTC 4)** | **-0.19** | **0.053** | **43** | | **-3.64** | **0.00073** |
|  | NSDS | 0.079 | 0.077 | 43 | | 1.03 | 0.3104 |
|  | WSC | 0.095 | 0.071 | 43 | | 1.35 | 0.1857 |
|  | WSDS | 0.080 | 0.074 | 43 | | 1.075 | 0.2885 |
|  | **8 DPI** | **-0.30** | **0.039** | **141** | | **-7.66** | **2.73 10^-12^** |
|  | **13 DPI** | **-0.13** | **0.039** | **141** | | **-3.37** | **0.000965** |
|  | **18 DPI** | **-0.11** | **0.039** | **141** | | **-3.00** | **0.003227** |
| WUE | **Intercept** | **4.43** | **0.17** | **96.41** | | **26.05** | **2.00 10^-16^** |
|  | **Variety (CTC 4)** | **-0.42** | **0.13** | **43** | | **-3.17** | **0.002818** |
|  | NSDS | 0.01 | 0.19 | 43 | | 0.076 | 0.939394 |
|  | WSC | 0.33 | 0.18 | 43 | | 1.87 | 0.06786 |
|  | WSDS | 0.13 | 0.19 | 43 | | 0.71 | 0.4833 |
|  | **8 DPI** | **0.60** | **0.17** | **141** | | **3.64** | **0.000384** |
|  | **13 DPI** | **0.77** | **0.17** | **141** | | **4.63** | **8.36 10^-6^** |
|  | **18 DPI** | **0.51** | **0.17** | **141** | | **3.05** | **0.002706** |
| A | **Intercept** | **30.64** | **1.11** | **86.67** | | **27.60** | **2.00 10^-16^** |
|  | **Variety (CTC 4)** | **4.27** | **0.90** | **43** | | **4.72** | **2.54 10^-5^** |
|  | **NSDS** | **-3.60** | **1.30** | **43** | | **-2.78** | **0.008099** |
|  | **WSC** | **-3.55** | **1.20** | **43** | | **-2.96** | **0.004934** |
|  | **WSDS** | **-3.58** | **1.26** | **43** | | **-2.85** | **0.006729** |
|  | **8 DPI** | **-2.92** | **1.02** | **141** | | **-2.86** | **0.004939** |
|  | **13 DPI** | **-3.81** | **1.02** | **141** | | **-3.73** | **0.000276** |
|  | **18 DPI** | **-4.27** | **1.02** | **141** | | **-4.18** | **5.07 10^-5^** |

**Supplementary Table 3.** ANOVA table from Mixed Linear Models (MLM) considering the effects of sugarcane varieties, biotic and abiotic stressors and time after sugarcane borer infestation (DPI) on gas exchange parameters

| Response* | Source of variation | Sum of squares | Mean square | df | F | P value | $\eta_{p}^{2}$** |
| --- | --- | --- | --- | --- | --- | --- | --- |
| Ci | **Variety** | **26487.0** | **26487.0** | **1** | **22.5623** | **2.29 10^-5^** | **0.34** |
|  | Treatment | 7177.5 | 2392.5 | 3 | 2.038 | 0.12 | 0.12 |
|  | **DPI** | **10137.4** | **3379.1** | **3** | **2.8784** | **0.04** | **0.06** |
| E | **Variety** | **1.72** | **1.72** | **1** | **36.62** | **3.04 10^-7^** | **0.46** |
|  | **Treatment** | **0.72** | **0.24** | **3** | **5.08** | **4.2 10^-3^** | **0.26** |
|  | **DPI** | **3.05** | **1.02** | **3** | **21.62** | **1.4 10^-11^** | **0.32** |
| gs | **Variety** | **4.25** | **4.25** | **1** | **57.97** | **1.68 10^-9^** | **0.57** |
|  | **Treatment** | **1.25** | **0.42** | **3** | **5.70** | **0.0022** | **0.28** |
|  | **DPI** | **2.25** | **0.75** | **3** | **10.26** | **3.75 10^-6^** | **0.18** |
| VPD | **Variety** | **0.49** | **0.49** | **1** | **13.24** | **7.2 10^-4^** | **0.24** |
|  | Treatment | 0.081 | 0.027 | 3 | 0.73 | 0.54 | 0.05 |
|  | **DPI** | **2.20** | **0.73** | **3** | **19.88** | **8.39 10^-11^** | **0.30** |
| WUE | **Variety** | **6.74** | **6.74** | **1** | **10.04** | **2.82 10^-3^** | **0.19** |
|  | Treatment | 2.85 | 0.95 | 3 | 1.41 | 0.25 | 0.09 |
|  | **DPI** | **16.03** | **5.34** | **3** | **7.96** | **6.12 10^-5^** | **0.14** |
| A | **Variety** | **492.83** | **492.83** | **1** | **17.85** | **1.21 10^-4^** | **0.29** |
|  | **Treatment** | **258.12** | **86.04** | **3** | **3.12** | **0.036** | **0.18** |
|  | **DPI** | **426.18** | **142.06** | **3** | **5.15** | **2.01 10^-3^** | **0.10** |

*Ci - CO_2_ Intercellular concentration; E - Transpiration; gs - stomach conductivity; VPD - vapor pressure deficit from the leaf to air; WUE - Water use efficiency in photosynthesis; A - assimilation/respiration.**$\eta_{p}^{2}$: Partial variance explained
